# The impact of consecutive COVID-19 lockdowns in England on mental wellbeing in people with inflammatory arthritis

**DOI:** 10.1186/s41927-022-00266-y

**Published:** 2022-06-29

**Authors:** Emma Caton, Hema Chaplin, Lewis Carpenter, Melissa Sweeney, Hsiu Yen Tung, Savia de Souza, James Galloway, Elena Nikiphorou, Sam Norton

**Affiliations:** 1 Health Psychology Section, Institute of Psychiatry, Psychology and Neuroscience, King’s College London, 5th Floor, Bermondsey Wing, Guy’s Hospital, London Bridge, London, SE1 9RT UK; 2 Centre for Rheumatic Diseases, King’s College London, London, UK

**Keywords:** COVID-19, Inflammatory arthritis, Qualitative, Mental health

## Abstract

**Background:**

During the first UK COVID-19 lockdown, studies identified over half of inflammatory arthritis (IA) patients in the UK reported a worsening of emotional distress. Given the prolonged nature of the pandemic, and the strict ‘shielding’ restrictions imposed on ‘extremely clinically vulnerable’ populations, it is likely that the implementation of the second lockdown period in England, during November 2020, may also have had a negative impact on the mental health of IA patients. The aim of this study was to qualitatively explore the impact of consecutive lockdown periods on mental wellbeing in people with IA.

**Methods:**

Nine IA patients took part in semi-structured telephone interviews at both baseline (June/July 2020) and follow-up (November 2020). The interview schedule, which was developed and piloted with a Patient Research Partner, explored patient experiences and mental health impacts of the COVID-19 lockdown periods. Interviews were analysed using inductive thematic analysis.

**Results:**

Five males and four females, with rheumatoid arthritis, psoriatic arthritis, or spondylarthritis, aged between 24–79 years (mean = 49.9, SD = 20.9) were included in the sample. Four main themes impacting on mental wellbeing were identified from the data: (1) Pandemic fatigue versus pandemic acclimatisation, (2) Social interaction and isolation, (3) Clarity of information, (4) Seasonal changes.

**Conclusion:**

The first two COVID-19 lockdown periods in England had an ongoing impact on the mental health of patients with IA. Healthcare professionals, in conjunction with government support, should ensure that adequate information and mental health resources are available to support IA patients during periods of ongoing restrictions, whilst also continuing to encourage behaviours which promote good mental health and wellbeing.

**Supplementary Information:**

The online version contains supplementary material available at 10.1186/s41927-022-00266-y.

## Background

Mental health comorbidities, such as anxiety and depression, are highly prevalent among patients with Inflammatory Arthritis (IA) [1, 2]. Poor mental health in this population has been linked to poor disease outcomes including increased pain [3], fatigue [4] and disease activity [5].

The COVID-19 pandemic has had a significant impact on emotional distress within the general population [6], with approximately a quarter of people in the UK experiencing moderate to severe levels of depressive symptoms during the first lockdown period [7]. During the pandemic, many IA patients were identified as being ‘extremely clinically vulnerable’ and were encouraged to follow strict ‘shielding’ guidelines [8]. Given people with IA are already susceptible to high levels of psychological distress [1, 2], it is possible that the COVID-19 pandemic may have had a substantial negative impact on their mental health. Research suggests that during the initial period of lockdown restrictions in the UK from March to July 2020, over half of IA patients studied reported a worsening of emotional distress [9]. Similar findings were also reported across Europe, with the prevalence of cases of anxiety and depression in patients with rheumatic and musculoskeletal conditions increasing beyond those identified in pre-pandemic studies [10].

Within the general population, high levels of psychological distress continued, and in some instances were observed to have worsened, during the second lockdown period, despite restrictions on movement and social interaction being less severe [11]. Researchers have speculated that the prolonged impact of the COVID-19 pandemic on mental health may be due to ‘pandemic fatigue’, which describes a feeling of mental exhaustion and demotivation to engage with recommended protective behaviours [12]. Despite this, it is also reasonable to surmise that psychological distress may have improved for some individuals during the second lockdown, as people became more familiar with the situation and were able to employ positive coping strategies. Nevertheless, little research has been conducted to compare the mental health experiences of IA patients between the first two lockdown periods, and the specific aspects of the second lockdown which may have contributed to mental health outcomes have yet to be explored. Therefore, the aim of this study was to qualitatively explore the impact of consecutive lockdown periods in England on the mental wellbeing of people with IA.

## Methods

### Design and recruitment

This qualitative study is nested within a larger mixed-methods longitudinal survey study (IA-COVID-19) exploring the impact of COVID-19 on health-related quality of life for people with IA.

Participants were recruited to the IA-COVID-19 study via social media and relevant charities. Criteria for inclusion were people over 18 years old, living in the UK and diagnosed with an IA condition (e.g. Rheumatoid Arthritis, Psoriatic Arthritis, Spondyloarthritis).

Following completion of the baseline survey for the main study, participants who provided consent for contact for additional research were invited to take part in the qualitative sub-study. Twenty-one participants took part in qualitative interviews between June and July 2020. These participants were then approached to take part in follow-up interviews in November 2020. Experiences from both the baseline and follow-up interviews were considered in relation to the second lockdown period to establish how participants’ experiences may have changed over time.

### Materials and procedure

Semi-structured interviews were conducted remotely via telephone during the second lockdown period in November 2020. An interview schedule (see Additional file 1: Table S1), which aimed to explore patient experiences of the second lockdown period, was developed with and piloted on a Patient Research Partner (SdS).

The interview schedule was adapted from the one used in the initial interviews in June and July. The adaptions included prompts about the events specific to the second lockdown, such as changes to shielding recommendations and the development of a COVID-19 vaccine, as well as a question focusing on how health-based messages could be better delivered to people with IA, following concerns expressed during the baseline interviews. The schedule consisted of ten main questions focusing on the impact of the second lockdown period on participants’ mental and physical health, self-management behaviours, healthcare access, COVID-19 vaccinations, and concerns for the future.

Patient interviews were conducted in English and lasted for approximately 15 min. The interviews were audio-recorded, with permission from participants, before being manually transcribed by the researcher (EC).

### Analysis

Data were analysed using inductive thematic analysis [13]. Inductive thematic analysis is a rigorous, data-driven approach which allows for a deep exploration into patient experiences. The transcripts were read and re-read by the lead researcher (EC) to ensure familiarity with the data, before being systematically coded. A randomly selected subset of transcripts (n = 2) were independently coded by another researcher (HC). Similar codes were collated and organised into themes. Themes were discussed with members of the research team (EC, HC, SN) to ensure that they were well-developed and representative of the data.

## Results

### Participants

A total of nine participants, from the original sample of 21 participants who were approached, agreed to take part in the qualitative interviews in November 2020 (see Table 1). The sample included five males and four females aged between 24 and 79 years old (mean = 49.9, SD = 20.9). Three IA condition were reflected in the sample (Psoriatic arthritis: *n* = 5; Spondyloarthritis: *n* = 3; Rheumatoid arthritis: *n* = 1).

**Table 1:** Participant demographics

Participant ID	Gender	Age	IA condition	Ethnicity	Geographical location
Participant 1	Female	70	Rheumatoid arthritis	White British	West Midlands
Participant 2	Female	35	Psoriatic arthritis	White British	South East England
Participant 3	Male	79	Psoriatic arthritis	White British	London
Participant 4	Male	72	Psoriatic arthritis	White British	London
Participant 5	Male	61	Psoriatic arthritis	White British	Yorkshire and the Humber
Participant 6	Male	46	Psoriatic arthritis	White British	South East England
Participant 7	Female	31	Spondyloarthritis	White British	London
Participant 8	Female	24	Spondyloarthritis	White British	East of England
Participant 9	Male	31	Spondyloarthritis	White British	South West England

### Themes

Four main themes were generated from the data to explain the impact of the first two consecutive lockdown periods in England on mental wellbeing in people with IA: (1) *Pandemic fatigue versus pandemic acclimatisation,* (2) *Social interaction and isolation,* (3) *Clarity of information,* (4) *Seasonal changes.* A full summary of the themes can be found in Table 2.

**Table 2:** Summary of themes

Themes	Description
Pandemic fatigue versus pandemic acclimatisation	The continuous nature of the pandemic and lockdown restrictions led to an increase in mental exhaustion and low mood for some participants during the second lockdown. Other participants expressed that their mental health improved during the second lockdown because they felt they had been able to acclimatise to the situation
Social interaction and isolation	Social interaction, including virtual communication, had a positive impact on mental health, whilst social isolation had a negative impact
Clarity of information	Clear information about COVID-19 and the recommended ‘shielding’ guidelines, helped to reduce emotional distress for IA patients A lack of clear information, either due to the limited amount of information available at the time or due to information being disseminated late, led to an increase in stress and anxiety for participants
Seasonal changes	Seasonal changes, such as poorer weather and darker days, led to some participants experiencing increased negative affect during the second lockdown

#### Theme 1: pandemic fatigue versus pandemic acclimatisation

Baseline interviews revealed that, for most IA patients, the first COVID-19 lockdown had a negative impact on their mental health:

*“The first six weeks were just horrific, like my moods were so low, my fatigue was bad anyway… I had no motivation to get up and do anything.”* (Participant 8, baseline)

*“Um, mental health aspect has probably been- that’s been more problematic… [the pandemic] just brought on a bit of, um, depression”* (Participant 5, baseline)

There was a divergence of impacts during the second lockdown, and a change in the nature of mental health symptoms experienced. When asked about their mental health during the second lockdown all participants, unprompted, compared their experience to those of the first lockdown period. Out of the nine participants interviewed, five expressed that their mental health worsened during the second lockdown; participants reported experiencing increased stress and low mood compared to the first lockdown period:

*“I’ve found it more harder [SIC] than the first lockdown to be honest… I found it more, kind of, depressing I suppose, made me feel bit low really going into the second lockdown.”* (Participant 6, follow-up)

*“I have to say in this lockdown period it was- it has been more difficult… I think it’s all building isn’t it, um, the continued lockdown is quite stressful I have to admit.”* (Participant 3, follow-up)

This suggests that the ‘continued lockdown’ may have taken a toll on IA patients, leading to increased negative affect for these individuals. Interestingly, the language used to describe mental health symptoms shifted from adjectives reflecting high levels of arousal, anxiety and agitation, to ones reflecting greater deactivation, more related to depression. For example, Participant 4 further illustrates that mental health may have further declined during the second lockdown as a result of people becoming ‘bored and tired’ of the continuous lockdown restrictions:

*“I think it’s [mental health] slightly dampened…[the lockdown’s] just sort of dragging on I think, you just sort of get bored and tired of it.”* (Participant 4, follow-up)

On the other hand, four of the nine participants reported that, compared to the first lockdown period, their mental health improved during the second lockdown:

*“[Mental health was] much better…the first lockdown was so severe, it was slightly surreal really, so yeah no it’s been- the second lockdown has been much better.”* (Participant 5, follow-up)

The phrase ‘slightly surreal’ suggests that it may have been the unusual and novel nature of the first lockdown that contributed to patients’ poor mental health, whereas in the second lockdown participants had a greater idea of what to expect. It was also noted that, as the pandemic continued, participants found it difficult to maintain high levels of anxiety:

*“I’m getting less depressed than I was in the first lockdown, um, and I don’t feel as anxious about things I guess…it’s been going on for such a long time it’s difficult to keep up that level of anxiety.”* (Participant 2, follow-up)

This suggests that the negative emotions arising from the pandemic may have naturally decreased over time as people adjusted and became accustomed to the COVID-19 situation. Nevertheless, it is important to note that none of the participants mentioned an improvement in their mental health beyond pre-pandemic levels, suggesting that improvement occurred in relation to reduced negative emotions, mostly associated with anxiety and agitation, rather than increased positive affect.

#### Theme 2: social interaction and isolation

During both lockdowns, most participants were able to maintain their social relationships by staying in regular contact with their family and friends virtually:

*“…not being able to see colleagues… we’ve adapted to that so we’ve actually seen each other more often, um, and have social drinking zooms [laughs] it- it’s quite good”* (Participant 9, baseline)

*“I make sure that I talk to [friends] every couple of weeks on like video calls and stuff…that’s something I still make time for in the evenings, so I have someone to, kind of, offload onto.”* (Participant 7, follow-up)

The term ‘someone to offload onto’ implies that communicating regularly with friends provided people with an opportunity to express their feelings and potentially relieve themselves of stress or worry. In addition to virtual communication, Participant 7 also expressed how returning to work during the second lockdown period allowed them to also have more face-to-face interactions:

*“I’m now back at work, I can talk to people a lot more, um, everyday, and I do feel really lucky that I get to actually go into a workplace rather than having to stay at home.”* (Participant 7, follow-up)

Participants who had returned to their workplace during the second lockdown noted that the increased social interactions greatly benefited their mental health:

*“…working in a small office between three or four of us, you know, we’re socially distancing so it’s nice to have the banter and the, um, connections… so from a mental point of view it’s been such an improvement.”* (Participant 5, follow-up)

In contrast, some participants felt that the lockdown restrictions impaired their ability to adequately socialise with their family and friends, especially in regard to meeting people face-to-face:

*“I was a bit sad that I wasn’t going to be able to see my friends inside… I think it was a bit disappointing”* (Participant 2, follow-up)

*“…the continued lockdown is quite stressful, I have to admit, I’d love to get and see my grandchildren in [Europe] but you know that’s not an option.”* (Participant 3, follow-up)

The terms ‘a bit sad’ and ‘quite stressful’ imply that restrictions on social interactions may have had a negative impact on the mental wellbeing of people who were unable to see their family and friends as a result. Nevertheless, participants who were living with their family members also reported low mood during the second lockdown period:

*“I did feel really isolated and a bit lonely actually, which is strange because I’m in quite a busy house, um [coughs], just kind of depressed and a bit low”* (Participant 6, follow-up)

This suggests that the interaction between social contact and mental wellbeing may be more complex than merely just considering the frequency of social interaction.

#### Theme 3: clarity of information

During the baseline interviews, several IA patients reported feeling high levels of stress and anxiety partly due to being identified as ‘extremely clinically vulnerable’ to severe illness from COVID-19:

*“I think being told you have, y’know, an autoimmune disease that you need to suppress your immune system…I kind of don’t even have the words…obviously I’m still here and everything’s ok but I’m not sure I have coped really.”* (Participant 2, baseline)

*“…obviously everything’s really scary and my own condition…I had lots of time to think, um, about kind of everything that could possibly happen”* (Participant 7, baseline)

Some participants noted that their anxieties about catching COVID-19, were further exacerbated by the lack of clear and specific information regarding how to behave during this time:

*“I think we’ve had so many mixed messages …and I think those mixed messages caused a great deal of, um, confusion and anxiety.”* (Participant 1, follow-up)

*“I got two letters, one in the first pandemic and now on the second pandemic, it just says we think you’re, um, we think you’re in a critical state, but it doesn’t say why…it didn’t say specifically about psoriatic arthriti*s.” (Participant 4, follow-up)

It is important to note that, due to the novelty of COVID-19, there was much uncertainty regarding its impact at the beginning of the pandemic, thus making it difficult for the government and healthcare professionals to disseminate high-quality advice. Nevertheless, some participants reported that, even when clear information was available, this was not always distributed to IA patients in a timely manner:

*“[My rheumatologist] informed me that I was, um, ‘shielding’, I didn’t actually hear officially from the government until some time later…and I found it very difficult, for instance, to get a supermarket delivery because of that”* (Participant 1, baseline)

*“There was no- there’s no communication tool and that- that I think caused a lot of, um, stress…I got a letter telling me that, you know, what risk I was… but that probably took two months for that letter to come through, and even just a text message would have been a way just to get a bit of information out, that would have been really helpful.”* (Participant 6, follow-up)

These quotes demonstrate how, in addition to causing stress and confusion for patients, the lack of an official ‘shielding’ letter for some individuals prevented them from seeking support services such as supermarket delivery slots, which may have also impacted upon their mental health. Indeed, Participant 6 suggests that disseminating information virtually via email or text messages may have been more effective than paper-based communication and could have potentially prevented some of the distress caused by the lack of timely communication.

Nevertheless, as the pandemic progressed and more information became available, participants noted that they had a greater understanding of the COVID-19 virus and its impact on society. This increased understanding is likely to have helped to reduce some of their mental distress, and may account for the reductions in symptoms of anxiety reported:

*“My anxiety has gone down…I think that comes with an understanding of what is going on”* (Participant 7, follow-up)

*“[Mental health is] better than it was in the first one [lockdown]…because we know more about the virus now”* (Participant 8, follow-up)

#### Theme 4: seasonal changes

Participants who expressed that their mental health worsened during the second lockdown suggested that this was partially due to the second lockdown being implemented during the winter months:

*“I must admit I'm finding it more difficult this second time, I think it's basically due to the time of year… we've got dark nights and dark mornings so it feels more restrictive this second time than it did first time.”* (Participant 1, follow-up)

*“…the lockdown is taking place at a time when days are getting darker, colder, lights dropping off, um, it’s a lot like going into a tunnel rather than on the first lockdown you were, even though it was bad, you were actually going towards summer warmth and long hot days”* (Participant 4, follow-up)

These quotes demonstrate how the weather had negatively impacted upon participants’ experiences of the second lockdown, making them feel more restricted and somewhat less optimistic compared to the first lockdown period. The relationship between seasonal changes and mental health in IA patients could be partly explained by the impact of weather patterns on physical symptoms, with some IA patients explaining that the poor weather had had a negative impact on their IA condition:

*“…if it's a really damp, horrible, cold, drizzly day, you- you get a little bit of something [worsening IA symptoms]”* (Participant 5, follow-up)

Additionally, the change in seasons may also have impacted upon patients’ abilities to engage in outdoor activities which usually benefit their mental health. For example, Participant 3 explained how being unable to utilise the garden during the winter months had a negative impact on his mental health:

*“I have to say in this lockdown period it was- it has been more difficult…the garden was a great relief in the last one but it it’s not so easy now there's less to do in the garden at this time of the year as well so, um yeah, it's been a little more stressful.”* (Participant 3, follow-up)

## Discussion

The purpose of this study was to explore the impact of consecutive lockdown periods on mental wellbeing in people with IA. Findings suggest that, compared to the first lockdown, some participants saw an improvement in their mental health, whilst for others their mental health worsened.

Given the significant disruptions to healthcare services during the pandemic, and the strict ‘shielding’ restrictions imposed on ‘extremely clinically vulnerable’ populations during the first lockdown, the researchers presumed that disease activity and IA-specific experiences would greatly influence participants’ mental health. For example, in a study exploring the impact of the first COVID-19 lockdown in people with rheumatic and musculoskeletal conditions, patients noted that the low mood associated with the pandemic and the anxiety of being identified as ‘clinically vulnerable’ had a negative impact on their disease activity [14]. Despite this, during the second lockdown, participants tended to speak more generally about their mental health rather than disclosing experiences specifically related to their IA condition. Indeed, the partial resumption of routine healthcare services at the end of the first lockdown may have given patients the opportunity to address their IA-specific concerns, thus reducing the prevalence of these issues during the second lockdown period.

In the current study, several participants attributed worsening mental health outcomes during the second lockdown to the ongoing nature of the pandemic and lockdown restrictions. In addition to suggesting that people may be experiencing a form of mental exhaustion in relation to the pandemic, these findings also support the idea that the COVID-19 lockdowns are likely to have a substantial negative impact on mental health outcomes in the long term [15]. Reassuringly, some participants reported better mental health during the second lockdown period, suggesting that they may have adapted to the challenges of the pandemic over time. Even so, it is important to note that participants only discussed the improvement in their mental health in relation to the first lockdown period. Thus, further research may be needed to establish how these improvements compare to pre-pandemic levels.

Social interaction was one factor identified as being beneficial for maintaining good mental health during the COVID-19 pandemic. Current literature suggests meaningful social interactions have a positive impact on mental health by cultivating feelings of self-worth and identity, and through embracing new technologies during the pandemic these interactions can continue to be established remotely [16]. As such, it is important that healthcare professionals promote the benefits of staying in contact with others, which may involve encouraging patients to embrace digital technologies, to prevent the negative impacts of social isolation on their mental health [17].

Another factor influencing the mental health of IA patients during the second lockdown was patient understanding and knowledge of COVID-19. Participants stated that having clear information and advice about COVID-19, helped reduce their emotional distress during the second lockdown. Conversely, patients who were identified as ‘extremely clinically vulnerable’ but did not receive any information regarding COVID-19, or who received information late, described how their mental health worsened as a result. It is important to note that due to the novelty of COVID-19, especially during the first lockdown, disseminating high-quality information and advice to the IA population was not immediately feasible. Nonetheless, findings from this study suggest that providing patients with up-to-date information may help improve mental health outcomes, so it is important to ensure that when such information is available, it is disseminated in a timely manner. Furthermore, participants recommended that in the future, more information could be delivered virtually to help prevent any potential delays in communication.

Seasonal changes were also a factor which appeared to influence participants’ mental health. Participants expressed that the winter weather made them feel more pessimistic which had a negative impact on their mood and mental health. Indeed, seasonal changes in mental health are not uncommon, with approximately 20% of the UK population experiencing “winter blues”; a mild form of Seasonal Affective Disorder [18]. In addition, research suggests that weather patterns such as low pressure, which is associated with high wind and precipitation, may be related to ‘high-pain events’ in people with chronic pain [19]. Whilst the link between physical symptoms and seasonal changes is heavily debated in relation to IA [20], it is possible that the winter months may have impacted patients’ physical symptoms which subsequently had a negative influence on their mental health. Consequently, healthcare providers such as the NHS need to consider deploying additional mental health resources, alongside adequate financial support from the UK government, to support individuals who may have high level of emotional distress following the winter lockdown period.

One limitation of the current study is the small sample size. The inclusion of only nine participants may have prevented thematic saturation from being achieved; potentially neglecting themes which may not have been represented in the current dataset. In addition, the use of social media to recruit participants may have potentially introduced bias into the study. Whilst social media has been identified as one of the most effective recruitment methods [21], targeting IA support groups and charities may have attracted individuals who were purposefully seeking support for their negative experiences of the pandemic. Despite this, the current sample included patients who had both positive and negative experiences of the second lockdown period thus diminishing this concern.

Another limitation of the study is the lack of geographical diversity. Given the introduction of a three-tier restriction system in October 2020, which saw different regions experience different lockdown restrictions according to their alert level [22], participants experiences of the pandemic are likely to vary according to their location. With the majority of the sample population living in Southern England, the results from this study may not be representative of experiences of people living outside this area during the pandemic.

The study also lacks ethnic diversity as all participants included in the sample were White British. As a result, findings from this study may not accurately reflect the experiences of IA patients from other ethnic backgrounds.

It is important to acknowledge that, whilst conducting the current study, the researchers had their own personal experiences of the COVID-19 pandemic, which may have influenced how the data were interpreted. In order to mitigate researcher bias, transcripts were coded systematically to ensure that key data items were not missed and regular discussions were conducted with members of the research team to ensure that all themes were derivative of the data.

Findings from this study suggest that the consecutive lockdown periods had a substantial impact on the mental health of IA patients. Future research should consider the potential long-term consequences of these changes on patients’ physical and mental health going forward. Overall, healthcare professionals, with the aid of financial and strategic support from the government and healthcare managers, should ensure that adequate mental health resources are allocated to support IA patients who may be struggling with their mental health following the pandemic, whilst also continuing to encourage behaviours promoting good mental health and wellbeing.

## Supplementary Information

**Additional file 1: Table S1.** Interview schedule

## Data Availability

Due to the transcripts containing information that could potentially allow for the identification of participants, the data that support the findings of this study are available from the corresponding author, EC, only upon reasonable request.

## Abbreviations

COVID-19: Coronavirus disease 2019
IA: Inflammatory arthritis
