# Supplementary material for: The impact of consecutive COVID-19 lockdowns in England on mental wellbeing in people with inflammatory arthritis
Source: BMC Rheumatol. 2022 Jun 29;6:37. doi: 10.1186/s41927-022-00266-y (PMC9241173; doi:10.1186/s41927-022-00266-y)
Supplement: Supplementary file 1 — Additional file 1: Table S1. Interview schedule [file 41927_2022_266_MOESM1_ESM.docx]

**Additional File 1**

| Supplementary Table 1. Interview schedule. |
| --- |
| *Interview schedule* |
| Have you experienced any COVID-19 symptoms? [If no, move to Q2, if yes, ask prompt questions]  Prompts: What symptoms did you experience? Have you been tested for COVID-19, and if so what was the result? Did you feel the need to self-isolate and why? Have these symptoms had an impact on your arthritis, and if so, how? |
| Could you tell me what life has been like since we last spoke in June/July?  Prompts: What was life like once the first lockdown/self-isolation period was over? What has life been like since the second lockdown was implemented? |
| How has your physical health been during the second lockdown?  Prompts: How has your arthritis been? Have you experienced any other symptoms? |
| How has your mental health has been during the second lockdown?  Prompts: How has it made you feel emotionally/in terms of your mood? |
| Have you continued with any of the behaviour changes you made regarding how you look after yourself on a daily basis? Have you made any new changes?  Prompts: Have you continued/made any changes to your diet or exercise behaviours? Are you still socially distancing or practicing increase hygiene measures e.g. regular handwashing, sterilising contact surfaces? Do you wear a face mask when you are going to be in a crowded or indoor space? Do you keep in contact with family/friends? |
| Have you experienced any ongoing or new disruptions to your usual IA healthcare and treatments?  Prompts: Have you have to continue consultations by telephone? What did you like/dislike about the telephone consultations? Any issues about taking or accessing your medications? How have you found adapting to these changes? |
| Do you have any concerns for the future following this period? |
| Do you think you will have a COVID-19 vaccine when it becomes available? |
| Do you have any advice for how messages to people with IA conditions (either from the government or your healthcare team) could be delivered more clearly in the future if a similar situation were to happen again? |
| Is there anything else you would like to add about your experiences during this period? |
